# Sex differences in loop gain measured via superimposed end‐expiratory breath holds and inspired steady‐state hypoxia

**DOI:** 10.1113/EP092285

**Published:** 2025-07-28

**Authors:** Benjamin W. L. MacKenzie, Nicole A. Johnson, Nicholas D. J. Strzalkowski, Trevor A. Day

**Affiliations:** ^1^ Department of Biology, Faculty of Science and Technology Mount Royal University Calgary Alberta Canada

**Keywords:** central sleep apnoea, hyperoxia, hypoxia, loop gain, respiratory chemoreflexes, sex differences

## Abstract

Acute low oxygen exposure (hypoxia) elicits a hypoxic ventilatory response (HVR), which increases ventilation and mitigates hypoxaemia. During sustained exposure to hypoxia, ventilatory acclimatization increases peripheral chemoreflex (HVR) sensitivity or chemoreflex loop gain (LG). Although increased ventilation protects oxygenation, increased LG contributes to ventilatory instability during sleep (central sleep apnoea; CSA). The HVR is highly variable between individuals, and the impact of sex on HVR variability and LG is unclear. At low altitude (1100 m), we aimed to characterize a novel breath hold method to quantify LG using a standardized series of short (∼10 s), voluntary, end‐expiratory breath holds (EEBH), using a background of steady‐state normobaric inspired hyperoxia (fraction of inspired oxygen; $F_{IO_{2}}$ = 1.0), normoxia (i.e., ambient air; $F_{IO_{2}}$ = 0.21) and hypoxia ($F_{IO_{2}}$ = 0.14; equivalent to ∼4300 m altitude). Further, we investigated sex differences in LG during differential oxygen exposure using this novel EEBH protocol to quantify LG. We hypothesized that (a) LG magnitude would follow an inverse oxygen‐dependent pattern with each inspired gas condition, and (b) that males would have higher LG than females. We recruited 36 healthy participants (18 females; 18 males). We found (a) a graded, inverse oxygen‐dependent effect on LG magnitude (*P *< 0.0001), and (b) a sex‐specific effect, whereby males had significantly larger LG magnitudes than females, but only in steady‐state inspired hypoxia (*P *< 0.032). Our EEBH protocol illustrates an intrinsic sex difference in chemoreflex LG in hypoxia, which may underlie known sex differences in CSA at high altitude and in heart failure populations.

## Abstract (highlights)

- **What is the central question of this study?**
  Using a novel breath hold method to quantify loop gain (LG) using a series of short (∼10 s), voluntary end‐expiratory breath holds (EEBH) and a randomized background of steady‐state hyperoxia (100% O_2_), normoxia (21% O_2_) and hypoxia (14% O_2_), are there sex‐differences in LG?
- **What is the main finding and its importance?**
  There was a graded, inverse oxygen‐dependent effect on LG magnitude, and a sex‐specific effect where LG was higher in males than females, but only in hypoxia. A novel end‐expiratory breath hold protocol revealed an intrinsic sex difference in chemoreflex LG with hypoxic exposure, which may underlie sex differences in central sleep apnoea at high altitude.

## 1 INTRODUCTION

The chemoreceptor control of breathing includes distinct central (brainstem) and peripheral (carotid body) chemoreflex feedback loops, driving increases or decreases in ventilation in response to transient changes in blood gases (O_2_ and CO_2_; Duffin, 2011; Guyenet & Bayliss, 2015). Specifically, acute low oxygen (hypoxia) drives a hypoxic ventilatory response (HVR), increasing ventilation and mitigating hypoxaemia. Furthermore, during ascent to high altitude (HA; >3000 m), ventilatory acclimatization increases peripheral chemoreflex sensitivity, or chemoreflex loop gain (LG; Ainslie et al., 2013; White et al., 1987). Although this increase in ventilation with acclimatization protects oxygenation (Ainslie et al., 2013), this increased LG contributes to ventilatory instability during sleep, driving the development of central sleep apnoea (CSA; e.g., Bird et al., 2023; Orr et al., 2017).

CSA is common with ascent to HA, increasing in severity with higher ascent and longer duration exposure (Ainslie et al., 2013; Bird et al., 2021; Patrician et al., 2024), and is associated with increases in respiratory chemoreflex LG (Javaheri and Badr, 2023). CSA is characterized by alternating periods (∼10–15 s) of excessive hyperventilation (over‐breathing) following a reduction (hypopnoea) or absence of airflow (apnoea) during sleep. In sleep studies, chemoreflex LG is defined as the ratio of the resulting ventilatory response subsequent to a reduction in ventilation (hypopnea or apnoea), representing the sensitivity of the respiratory chemoreflex feedback loop in response to a ventilatory disturbance (i.e., stimulus; Javaheri & Dempsey, 2013; Wellman et al., 2008). These values are quantified from the respiratory flow signal from the nasal canula using polysomnography systems and/or portable sleep monitors (Bird et al., 2023; Wellman et al., 2008). LG values over 1.0 represent susceptibility to ventilatory instability during sleep and CSA (Javaheri & Dempsey, 2013).

Testing respiratory chemoreflexes using traditional lab‐based steady‐state methods (e.g., Steinback & Poulin, 2007) may not be relevant to CSA, given the transient nature of oscillations in apnoea‐hyperventilation cycles. In addition, they lack feasibility to predict those susceptible to CSA, particularly in clinical contexts or HA fieldwork expeditions. Voluntary breath holds, with their associated transient changes in blood gases associated with the metabolic rate, have been used to stimulate respiratory chemoreflexes and calculate LG during wakefulness (e.g., Messineo et al., 2018), but have not been employed in a background of different gases, nor with ascent to HA. The HVR magnitude is highly variable between individuals (e.g., Fatemian et al., 2016; Oeung et al., 2023), and the potential impact of biological sex on LG is equivocal (Raberin et al., 2024). There are inconsistencies in reports of sex differences in the HVR, where they have been previously reported as greater in females (Aitken et al., 1986), greater in males (Goldberg et al., 2017; White et al., 1983), or are similar between sexes (Guenette et al., 2004; Macnutt et al., 2012; Rispen et al., 2017). However, Bird et al. (2023) demonstrated that males have a higher LG during sleep, and thus increased incidence and severity of CSA, compared to females over a 9–10 day acclimatization period at 3800 m. It remains unclear if this sex difference in LG is apparent in a background of alterations in steady‐state normobaric oxygen levels, namely hyperoxia, normoxia and/or hypoxia, following voluntary breath holds during wakefulness.

Here, we aimed to develop and characterize an alternative, novel, voluntary, end‐expiratory breath hold (EEBH) protocol to quantify chemoreflex LG (a) to simulate the CSA stimulus during wakefulness, and (b) as a potential tool to assess chemoreflex responsiveness (i.e., LG), in both laboratory and fieldwork studies. Specifically, we utilized a standardized series of short (∼10 s), voluntary EEBHs, using a background of acute inspired hyperoxia (fraction of inspired oxygen; $F_{IO_{2}}$ = 1.0; to partly silence the carotid bodies), normoxia (i.e., ambient air; $F_{IO_{2}}$ = 0.21; control) and hypoxia ($F_{IO_{2}}$ = 0.14; ∼equivalent to 4300 m; to sensitize the carotid bodies). We hypothesized that (a) LG will be lower, intermediate and higher, within‐individual, respectively, following each series of voluntary breath holding in each inspired steady‐state gas challenge, and that (b) there will be a sex‐specific difference in chemoreflex LG, with males being higher than females.

## 2 METHODS

### 2.1 Ethical clearance

This study was approved in advance by Mount Royal University (MRU) Human Research Ethics Board (Protocol #103712). Participant recruitment and the data collection protocol were consistent with the Canadian Government Tri‐Council Policy on human research participants and the *Declaration of Helsinki*, except for registration in a database.

### 2.2 Participant recruitment

We recruited a large convenience sample of both healthy male (*n* = 18) and healthy female (*n* = 18) participants by verbal communication. All participants were between 18 and 35 years of age, with a body mass index (BMI) of less than 30 kg/m^2^, with males and females matched for age and BMI (see Results). They were free from taking prescription drugs, aside from oral contraceptives, were non‐smokers, and had no known or reported existing cardiovascular, respiratory or neurological diseases at the time of the study. The ovarian cycle was not considered in the recruitment criteria of females for this study. Participants were asked to refrain from ingesting any caffeinated or alcoholic beverages and engaging in vigorous activity for 12 h before the experimental testing. Recruitment and data collection occurred in a single laboratory visit (MRU Integrative Physiology Laboratory; 1100 m). Participants provided free, informed, verbal and written consent before voluntary participation in the study.

### 2.3 Instrumentation and data collection

Participants were positioned in a supine position on a bed for instrumentation and throughout data collection. Data were collected using a 16‐channel PowerLab system (PowerLab 16/35 ML880; ADInstruments (ADI), Colorado Springs, CO, USA) and analysed offline using LabChart Pro 8.0 (ADInstruments). Participants were instrumented with a lead II electrocardiogram (ECG; ADI bioamp ML132) and finger photoplethysmography placed on the right middle finger (Finometer Nova, Finapres Medical Systems, Enschede, the Netherlands). Heart rate (HR) was calculated as 60/period from the raw ECG signal. Both mean arterial pressure (MAP) and systolic blood pressure (SBP) were derived from the raw Finometer waveform. Peripheral oxygen saturation ($S_{pO_{2}}$; %) was measured by a peripheral pulse oximeter (ADI ML320; ADInstruments) placed on the left middle finger. Respiratory flow was measured by a pneumotachometer (HR 800L flow head and spirometer amplifier, 3813 series, Hans Rudolph, Shawnee, KS, USA; and ADI ML141; ADInstruments; calibrated daily with a 3‐L syringe). Inspired minute ventilation (*V̇*_I_; L/min) was determined as the product of breath‐by‐breath inspired tidal volume (*V*_TI_; calculated from the integral of the flow signal) and respiratory rate (RR; min^−1^; calculated by 60/period of the flow signal). Ventilatory drive was calculated as tidal volume (*V*_T_; L) divided by inspiratory time (*T*_I_; seconds) of a single breath. The breathing apparatus used to measure breath‐by‐breath ventilation included a nose‐clip, disinfected mouthpiece, personal disposable bacteriological filter, and a gas sampling port, proximal to the mouth, to sample and measure O_2_ and CO_2_ percentages continuously using a dual gas analyser, calibrated daily with known concentrations of O_2_ and CO_2_ (ADI ML206; ADInstruments). The breathing apparatus also incorporated a three‐way valve to allow switching of airflow between room air and two 200 L Douglas bags prefilled with either 100% O_2_ or 14% O_2_ (balance N_2_) from known gas tanks (Linde Canada Inc., Ontario, Canada). End‐tidal partial pressures of O_2_ and CO_2_ ($P_{\mathrm{ET}O_{2}}$; $P_{\mathrm{ETC}O_{2}}$; mmHg) were measured and corrected for body temperature and pressure, saturated with water vapor (BTPS; ∼660 mmHg *P*_ATM_ in Calgary, ∼1100 m) using daily atmospheric pressure measurements.

### 2.4 Protocol

**FIGURE 1:**
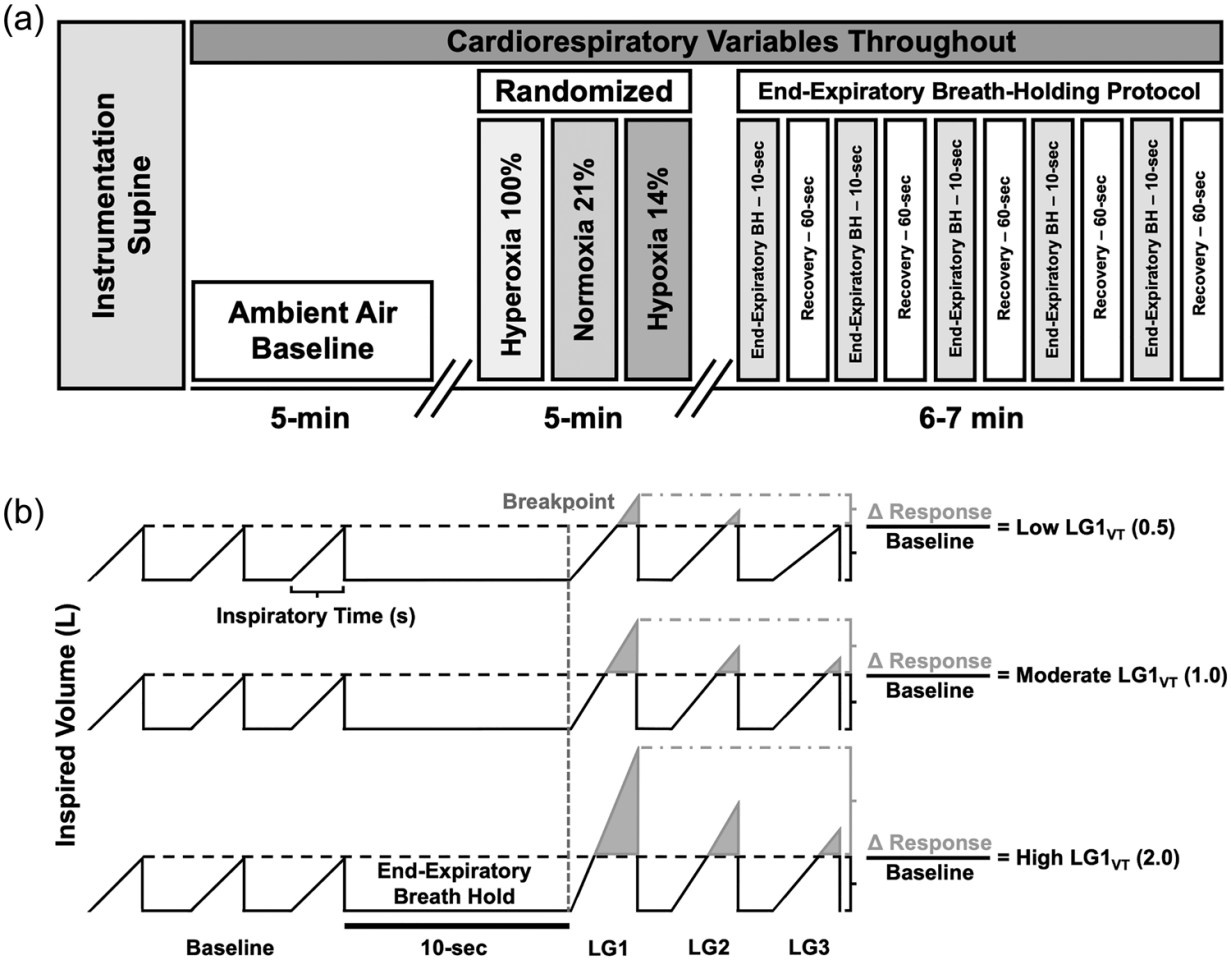
Schematic representation of the protocol and analysis. (a) Participants underwent a preliminary ambient air baseline for 5 min and then were exposed to three separate steady‐state inspired gases in a randomized order for 5 min: $F_{IO_{2}}$ 1.00, 0.21 (i.e., ambient air) and 0.14. They were then coached through a series of five 10 s end‐expiratory breath holds (EEBH), followed by 60 s recovery. Following the last recovery, the participants were exposed to the next randomized inspired gas, and the EEBH protocol was repeated. Each breath hold protocol in each gas took ∼16 min, with a total protocol time of ∼50 min. Cardiorespiratory variables were measured throughout (see Methods). (b) Analytical method for calculating loop gain (LG_VT_ or LG_VT/TI_) following a 10 s voluntary end‐expiratory breath hold (EEBH). Three inspired tidal volume (*V*_TI_; L) traces are shown to represent three theoretical magnitudes of LG. Following a 10 s EEBH, the delta response (in grey) is highlighted, which was standardized to a 15 s average baseline (BL) bin (dashed black line; i.e., response over stimulus). To note, LG_VT_ uses only the delta *V*_TI_ standardized to the BL bin, whereas LG_VT/TI_ uses the delta *V*_TI_ divided by inspiratory time (*T*_I_; seconds) as an index of central respiratory drive, which is also standardized to the 15 s BL bin. The top row demonstrates low LG1_VT_, with a ratio of 0.5 (i.e., delta response is 1/2 of BL). The middle row indicates moderate LG1_VT_, with a ratio of 1.0 (i.e., delta response is the same as BL). The bottom row exhibits high LG1_VT_, with a ratio of 2.0 (delta response is double that of BL). LG1_VT_, loop gain ratio using relative delta inspired tidal volume of the first breath following the breakpoint. LG1, loop gain ratio using the first breath following the breakpoint; LG2, loop gain ratio using the second breath following the breakpoint; LG3, loop gain ratio using the third breath following the breakpoint.

Following instrumentation, an initial 5 min baseline period breathing ambient air (i.e., normoxia) took place. Participants then underwent a secondary 5 min baseline period when exposed to one of three steady‐state inspired gas conditions: inspired hyperoxia ($F_{IO_{2}}$ = 1.0), normoxia (i.e., ambient air; $F_{IO_{2}}$ = 0.21) and hypoxia ($F_{IO_{2}}$ = 0.14) in a within‐individual randomized order. In each subsequent inspired gas baseline, participants were coached through five consecutive voluntary ∼10 s EEBHs at functional residual capacity, separated by ∼60 s recovery, for a total of ∼16 min per inspired gas condition. The apnoea duration of 10 s was chosen as this is more similar in duration to a central apnoea event (see below). Previous pilot testing demonstrated that participants’ *V̇*_I_, *V*_T_ and *V*_T_/*T*_I_ were fully recovered by 1 min following each breath hold back to baseline values. The protocol was repeated with the remaining gas conditions in a randomized order. The $F_{IO_{2}}$ 0.14 step is approximately equivalent to the partial pressure of inspired oxygen at ∼4300 m altitude, an intermediate HA where field studies assessing CSA have been carried out by our group (e.g., Bird et al., 2021). The total protocol time was approximately 50 min (see Figure 1a).

### 2.5 Calculations and data analysis

To better simulate CSA and to approximate the AASM criteria to score an apnoea (10 s), we modified the breath hold protocol from Messineo et al. (2018), which utilized end‐inspiratory apnoeas and a 20 s duration. Specifically, we modified this protocol to (a) utilize end‐expiratory apnoeas (breath holds), as this better simulates the apnoeas associated with CSA (Cao et al., 2013; Dempsey et al., 2010), and (b) reduce the duration of the voluntary apnoeas to ∼10 s, as this is the minimum apnoea duration scored using the American Academy of Sleep Medicine criteria (AASM).

We used this novel method of a series of five consecutive voluntary EEBHs to calculate LG ratios. Specifically, a 2 min baseline bin at the end of each baseline period was quantified for all participants to assess baseline cardiorespiratory variables in each inspired gas. Analysis included voluntary (but coached) breath hold duration (in seconds), recovery (from breath hold) duration (in seconds), baseline (BL) $S_{pO_{2}}$ (15 s mean bin prior to the start of the breath hold), nadir $S_{pO_{2}}$ (the minimum value during each recovery period following a breath hold), and delta $S_{pO_{2}}$ (subtracting the nadir $S_{pO_{2}}$ from the BL $S_{pO_{2}}$).

LG_VT_ was quantified as the delta *V*_T_ of the first three breaths following the coached breath hold, with each breath individually normalized to a 15 s BL bin prior to the start of each breath hold, then indexed against the BL bin (i.e., relative difference). Three steps were used: (a) a 15 s mean *V*_TI_ BL bin was selected prior the breath hold; (b) we subtracted this 15 s mean *V*_TI_ BL bin from each peak *V*_T_ of the breaths following the breath hold to calculate the delta (i.e., response); and (c) we divided the delta *V*_T_ of each breath following breakpoint (i.e., the stimulus, the magnitude of the reduction to apnoea) against our 15 s mean *V*_TI_ BL bin to obtain our LG_VT_ ratio (see Figure 1b). LG_VT_ ratios were then quantified using the first, average of first + second, second and third breath following each breath hold. Each ratio is denoted by an abbreviation: LG1_VT_ is LG_VT_ using the first breath following breakpoint; LG1+2_VT_ is LG_VT_ using an average of the first and second breath following breakpoint; LG2_VT_ is LG_VT_ using the second breath following breakpoint; and LG3_VT_ is LG_VT_ using the third breath following breakpoint. Each ratio averaged five breath hold values to obtain a representative, within‐individual LG_VT_ value.

LG_VT/TI_ was quantified in a similar way to LG_VT_. The delta ventilatory drive (*V*_T_/*T*_I_) of the first three breaths following the coached breath hold was individually normalized to a BL bin containing the mean *V*_T_/*T*_I_ for three breaths prior to the start of each breath hold (i.e., relative difference). Three steps were used: (a) *V*_T_/*T*_I_ was calculated and averaged over three breaths prior to the start of each breath hold to establish the *V*_T_/*T*_I_ BL bin; (b) we then calculated the delta *V*_T_/*T*_I_ by subtracting the individual breaths (first, second, third) post‐breath hold from the *V*_T_/*T*_I_ BL bin (i.e., response); and (c) we normalized the delta *V*_T_/*T*_I_ of the individual breaths following the breath hold to the *V*_T_/*T*_I_ BL bin to arrive at our LG_VT/TI_ ratio (see Figure 1b). LG_VT/TI_ ratios were then quantified using the first, average of first + second, second and third breath following each breath hold to mirror LG_VT_ ratios. Each ratio averaged five breath‐hold values to obtain a representative, within‐individual LG_VT/TI_ value.

### 2.6 Statistical analysis

All cardiorespiratory data are represented as means ±SD in table format. To assess changes in continuous cardiorespiratory variables, including breath hold variables across each inspired gas for the group, statistical analysis included a one‐factor repeated‐measures ANOVA. Where significant *F*‐ratios were detected, Tukey's *post hoc* test was utilized for pairwise comparisons.

To assess changes in continuous cardiorespiratory variables, including breath hold variables across each inspired gas, sex and interaction, statistical analysis included a two‐factor mixed model ANOVA (repeated‐measures in gas, non‐repeated‐measures in sex). Where significant *F*‐ratios were detected, Tukey's *post hoc* test was utilized for pairwise comparisons.

To compare both LG_VT_ and LG_VT/TI_ ratios for the three breaths following the breath hold across three inspired gas conditions for the group, we utilized a one‐factor repeated‐measures ANOVA. Where significant *F*‐ratios were detected, Tukey's *post hoc* test was utilized for pairwise comparisons.

To compare sex differences across both LG_VT_ and LG_VT/TI_ ratios and different $F_{IO_{2}}$, we used Student's unpaired *t*‐test using Welch's correction. In all cases, statistical significance was assumed at *P *< 0.05 (GraphPad Prism v10.0, GraphPad Software, Boston, MA, USA).

## 3 RESULTS

Data are presented as means ± standard deviation (SD) in text, tables and figures.

### 3.1 Participant demographics

We recruited 36 healthy participants (22.4 ± 2.0 years old; BMI = 24.0 ± 3.2 kg/m^2^), which included 18 males (22.9 ± 2.0 years old; BMI = 24.4 ± 3.4 kg/m^2^) and 18 females (21.8 ± 1.9 years old; BMI = 23.6 ± 3.2 kg/m^2^) that were included in the final data analysis. One female participant was excluded from the nadir $S_{pO_{2}}$ and delta $S_{pO_{2}}$ analyses due to her $S_{pO_{2}}$ dropping to the floor of our pulse oximeter model in hypoxia (70%), leaving 18 males and 17 females included for these variables (Tables 1 and 3).

**TABLE 1:** Baseline cardiorespiratory and breath hold variables across three gas conditions ($F_{IO_{2}}$ 0.14, 0.21, 1.00; *n* = 36).

Variable	$F_{IO_{2}}$ = 1.0	$F_{IO_{2}}$ = 0.21	$F_{IO_{2}}$ = 0.14	*P*
Baseline cardiorespiratory variables				
$F_{IO_{2}}$ (mmHg)	98.2 ± 1.7^b^	20.9 ± 0.2	14.1 ± 0.4^b^	**<0.0001**
HR (min^−1^)	62.0 ± 10.4^b^	67.1 ± 10.6	77.9 ± 12.4^b^	**<0.0001**
MAP (mmHg)	89.3 ± 7.9	88.1 ± 9.7	91.9 ± 9.0^b^	**0.0004**
SBP (mmHg)	121.9 ± 10.6	120.2 ± 12.8	123.8 ± 11.4^b^	**0.015**
$S_{pO_{2}}$ (%)	99.7 ± 0.4^b^	97.0 ± 1.5	85.2 ± 3.8^b^	**<0.0001**
RR (bpm)	13.4 ± 3.8	12.8 ± 3.8	13.3 ± 4.1	0.25
*V*_TI_ (L)	0.96 ± 0.27^b^	0.90 ± 0.23	0.96 ± 0.22^b^	**0.016**
*V̇*_I_ (L/min)	12.0 ± 2.0^b^	10.8 ± 1.8	12.2 ± 2.1^b^	**0.008**
$P_{\mathrm{ETC}O_{2}}$ (mmHg)	33.7 ± 2.8^b^	35.7 ± 2.8	33.5 ± 2.2^b^	**<0.0001**
$P_{\mathrm{ET}O_{2}}$ (mmHg)	555.7 ± 15.7^b^	83.5 ± 6.4	50.4 ± 2.9^b^	**<0.0001**
Breath‐holding variables				
LG1_VT_ (a.u.)	0.35 ± 0.46^b^	0.69 ± 0.53	1.00 ± 0.69^b^	**<0.0001**
LG1+2_VT_ (a.u.)	0.24 ± 0.27^b^	0.52 ± 0.34	0.69 ± 0.43^b^	**<0.0001**
LG2_VT_ (a.u.)	0.12 ± 0.20^b^	0.35 ± 0.34	0.37 ± 0.31	**<0.0001**
LG3_VT_ (a.u.)	0.10 ± 0.17	0.16 ± 0.24	0.12 ± 0.22	0.23
LG1_VT/TI_ (a.u.)	0.28 ± 0.35^b^	0.63 ± 0.47	1.22 ± 1.07^b^	**<0.0001**
LG1+2_VT/TI_ (a.u.)	0.18 ± 0.21^b^	0.46 ± 0.32	0.84 ± 0.72^b^	**<0.0001**
LG2_VT/TI_ (a.u.)	0.07 ± 0.15^b^	0.29 ± 0.25	0.45 ± 0.43	**<0.0001**
LG3_VT/TI_ (a.u.)	0.07 ± 0.13^b^	0.17 ± 0.23	0.16 ± 0.23	**0.035**
*T*_I_ 1 (s)	2.05 ± 0.56	2.00 ± 0.49	1.86 ± 0.55	**0.013**
*T*_I_ 1+2 (s)	2.04 ± 0.58	2.03 ± 0.60	1.92 ± 0.67	0.074
*T*_I_ 2 (s)	2.03 ± 0.61	2.06 ± 0.71	1.97 ± 0.78	0.47
*T*_I_ 3 (s)	2.01 ± 0.58	1.97 ± 0.59	2.01 ± 0.84	0.79
Breath hold duration (s)	10.1 ± 0.4	10.1 ± 0.3	10.0 ± 0.4	0.2
Recovery duration (s)	64.3 ± 3.3	65.0 ± 3.2	65.2 ± 3.1	0.11
BL $S_{pO_{2}}$ (%)	99.7 ± 0.4^b^	96.5 ± 2.9	83.8 ± 4.7^b^	**<0.0001**
Nadir $S_{pO_{2}}$ (%)	99.5 ± 0.6^b^	94.9 ± 3.2	79.5 ± 4.6^a^, ^b^	**<0.0001**
Delta $S_{pO_{2}}$ (%)	0.30 ± 0.37^b^	1.72 ± 0.70	4.77 ± 2.44^a^, ^b^	**<0.0001**

*Note*: *P*‐value represents the overall 1F RM ANOVA between $F_{IO_{2}}$ (0.14, 0.21, 1.00; *n* = 36). We bolded those hat were significant (*P* < 0.05).

^a^ Reduced sample size, *n* = 35.

^b^ Significantly different from $F_{IO_{2}}$ 0.21. Baseline cardiorespiratory variables: $F_{IO_{2}}$, fraction of inspired oxygen (%); HR, heart rate (min^−1^); MAP, mean arterial pressure (mmHg); SBP, systolic blood pressure (mmHg); $S_{pO_{2}}$, peripheral saturation of oxygen (%); RR, respiratory rate (bpm); *V*_TI_, inspired tidal volume (L); *V̇*_I_, inspired minute ventilation (L/min); $P_{\mathrm{ETC}O_{2}}$, partial pressure of end‐tidal carbon dioxide (mmHg); $P_{\mathrm{ET}O_{2}}$, partial pressure of end‐tidal oxygen (mmHg). Breath holding variables: LG_VT_, loop gain using relative delta tidal volume of the 1st breath, average of the 1st and 2nd breath, 2nd breath, 3rd breath following breath hold (a.u.); LG_VT/TI_, loop gain using relative delta respiratory drive of the 1st breath, average of the 1st and 2nd breath, 2nd breath, 3rd breath following breath hold (a.u.); *T*_I_, time of inspiration (s) of the 1st breath, average of the 1st and 2nd breath, 2nd breath, 3rd breath following breath hold; BL $S_{pO_{2}}$, baseline $S_{pO_{2}}$ prior to start of breath hold (%); Nadir $S_{pO_{2}}$, minimum $S_{pO_{2}}$ during recovery (%); Delta $S_{pO_{2}}$, difference of BL $S_{pO_{2}}$ and nadir $S_{pO_{2}}$.

### 3.2 Baseline cardiorespiratory and breath‐holding variables

Baseline cardiorespiratory variables and breath holding variables were obtained at all three $F_{IO_{2}}$ levels (i.e., 1.00, 0.21 and 0.14) and are reported in Table 1. Of note, during baseline conditions, there were significant decreases and increases (*P *< 0.016) for all variables except RR and SBP across hyperoxia, normoxia and hypoxic trials. These responses confirm that the hypoxic and hyperoxic stimuli were significantly different from normoxia. During the breath‐holding section, all of the variables were different across conditions (*P *< 0.035) except for breath‐hold duration, recovery duration, LG3_VT_, and LG3_TI_.

Metrics of continuous cardiorespiratory variables differing by sex across the three $F_{IO_{2}}$ levels are reported in Table 2, with main effects (gas and sex) and interactions reported. There were significant main effects of sex across all three gas conditions with $S_{pO_{2}}$, *V̇*_I_, $P_{\mathrm{ET}O_{2}}$ and $P_{\mathrm{ETC}O_{2}}$ (*P *< 0.045; Table 2). $S_{pO_{2}}$ was slightly higher in females compared to males in normoxia (*P* = 0.045). There were elevations observed in *V̇*_I_ for both hypoxia and hyperoxia compared to normoxia (*P *< 0.0001), but males had a larger magnitude of *V̇*_I_ than females in hypoxia (*P* = 0.039). Males also had a consistently higher $P_{\mathrm{ETC}O_{2}}$ than females across all conditions (*P* = 0.006), whereas the opposite was seen in $P_{\mathrm{ET}O_{2}}$, with females having a larger magnitude than males in normoxia and hypoxia (*P* = 0.014).

**TABLE 2:** Sex differences in baseline cardiorespiratory variables across three gas conditions ($F_{IO_{2}}$ 0.14, 0.21, 1.00; males *n* = 18, females *n* = 18).

		Sex		*P*		
Variable	$F_{IO_{2}}$	Females	Males	Sex (^c^)	Gas (^b^)	Interaction
$F_{IO_{2}}$ (mmHg)	1.00	98.2 ± 1.8^b^	98.2 ± 1.5^b^	0.93	**<0.0001**	0.96
	0.21	20.9 ± 0.2	20.9 ± 0.2			
	0.14	14.1 ± 0.3^b^	14.1 ± 0.4^b^			
HR (min^−1^)	1.00	64.0 ± 10.2^b^	60.0 ± 10.5^b^	0.38	**<0.0001**	0.35
	0.21	69.1 ± 10.1	65.1 ± 11.0			
	0.14	78.7 ± 12.9^b^	77.2 ± 12.3^b^			
MAP (mmHg)	1.00	90.0 ± 8.3	88.5 ± 7.6	0.8	**0.0005**	0.51
	0.21	87.8 ± 9.3	88.4 ± 10.4			
	0.14	92.6 ± 10.3^b^	91.3 ± 7.7			
SBP (mmHg)	1.00	120.6 ± 10.2^b^	123.2 ± 11.1	0.32	**0.016**	0.56
	0.21	117.7 ± 11.1	122.8 ± 14.1			
	0.14	122.2 ± 11.8^b^	125.5 ± 11.2			
$S_{pO_{2}}$(%)	1.00	99.8 ± 0.4^b^	99.7 ± 0.4^b^	**0.045**	**<0.0001**	0.19
	0.21	97.7 ± 1.3^c^	96.4 ± 1.5			
	0.14	86.1 ± 4.3^a^, ^b^	84.2 ± 3.0^b^			
RR (bpm)	1.00	12.9 ± 4.6	13.8 ± 3.0	0.35	0.26	0.88
	0.21	12.1 ± 4.0	13.5 ± 3.7			
	0.14	12.7 ± 3.8	13.9 ± 4.3			
*V*_TI_ (L)	1.00	0.97 ± 0.28	0.94 ± 0.25	0.93	**0.015**	0.19
	0.21	0.91 ± 0.24	0.88 ± 0.23			
	0.14	0.94 ± 0.20	0.99 ± 0.24^b^			
*V̇*_I_ (L/min)	1.00	11.5 ± 1.9^b^	12.5 ± 2.0^b^	**0.039**	**<0.0001**	0.34
	0.21	10.3 ± 1.8	11.2 ± 1.7			
	0.14	11.4 ± 1.7^b^, ^c^	13.0 ± 2.1^b^			
$P_{\mathrm{ETC}O_{2}}$ (mmHg)	1.00	32.6 ± 2.0^b^, ^c^	34.9 ± 3.0^b^	**0.006**	**<0.0001**	0.07
	0.21	34.4 ± 2.7^c^	37.1 ± 2.3			
	0.14	32.7 ± 2.1^b^, ^c^	34.3 ± 2.2^b^			
$P_{\mathrm{ET}O_{2}}$ (mmHg)	1.00	560.2 ± 14.9^b^	551.2 ± 15.6^b^	**0.014**	**<0.0001**	0.27
	0.21	85.9 ± 5.8^c^	81.1 ± 6.2			
	0.14	51.5 ± 3.0^b^, ^c^	49.4 ± 2.5^b^			

*Note*: *P*‐values are split between the sex effect, gas (oxygen) effect, and the interaction between sex and gas, from a two factor (repeated measures in gas; non‐repeated measures in sex) ANOVA across $F_{IO_{2}}$ (0.14, 0.21, 1.00) for each respective sex (males *n* = 18; females = 18). We bolded those hat were significant (*P* < 0.05)

^a^ Reduced sample size, *n* = 17.

^b^ Significantly different from $F_{IO_{2}}$ 0.21.

^c^ Significantly different from males. Baseline cardiorespiratory variables: $F_{IO_{2}}$, fraction of inspired oxygen (%); HR, heart rate (min^−1^); MAP, mean arterial pressure (mmHg); SBP, systolic blood pressure (mmHg); $S_{pO_{2}}$, peripheral saturation of oxygen (%); RR, respiratory rate (bpm); *V*_TI_, inspired tidal volume (L); *V̇*_I_, inspired minute ventilation (L/min); $P_{\mathrm{ETC}O_{2}}$, partial pressure of end‐tidal carbon dioxide (mmHg); $P_{\mathrm{ET}O_{2}}$, partial pressure of end‐tidal oxygen (mmHg).

Breath‐holding metrics that differ by sex across the three $F_{IO_{2}}$ levels are reported in Table 3, in which females had shorter breath‐hold durations compared to males across all conditions (*P* = 0.0052). BL, nadir and delta $S_{pO_{2}}$ were all different across conditions (*P *< 0.0001), but there were no differences between males and females for each $S_{pO_{2}}$ variable (*P *> 0.11).

**TABLE 3:** Sex differences in breath hold variables across three of the gas conditions ($F_{IO_{2}}$ 0.14, 0.21, 1.00; males *n* = 18, females *n* = 18).

		Sex		*P*		
Variable	$F_{IO_{2}}$	Females	Males	Sex (^c^)	Gas (^b^)	Interaction
LG1_VT_ (a.u.)	1.00	0.23 ± 0.27^b^	0.50 ± 0.53^b^	**0.046**	**<0.0001**	**0.044**
	0.21	0.59 ± 0.39	0.72 ± 0.62			
	0.14	0.74 ± 0.49^c^	1.26 ± 0.69^b^			
LG1+2_VT_ (a.u.)	1.00	0.16 ± 0.16^b^	0.32 ± 0.33^b^	**0.023**	**<0.0001**	0.067
	0.21	0.43 ± 0.30	0.55 ± 0.38			
	0.14	0.50 ± 0.33^c^	0.86 ± 0.44^b^			
LG2_VT_ (a.u.)	1.00	0.09 ± 0.13	0.15 ± 0.26^b^	0.084	**<0.0001**	0.31
	0.21	0.26 ± 0.32	0.38 ± 0.36			
	0.14	0.25 ± 0.25^c^	0.47 ± 0.32			
LG3_VT_ (a.u.)	1.00	0.09 ± 0.12	0.09 ± 0.21	0.28	0.28	0.16
	0.21	0.12 ± 0.21	0.18 ± 0.27			
	0.14	0.04 ± 0.13	0.18 ± 0.27			
LG1_VT/TI_ (a.u.)	1.00	0.24 ± 0.24^b^	0.31 ± 0.43^b^	0.25	**<0.0001**	0.17
	0.21	0.62 ± 0.37	0.63 ± 0.57			
	0.14	0.97 ± 0.50^b^	1.47 ± 1.41			
LG1+2_VT/TI_ (a.u.)	1.00	0.16 ± 0.14^b^	0.19 ± 0.27^b^	0.18	**<0.0001**	0.17
	0.21	0.44 ± 0.26	0.48 ± 0.39			
	0.14	0.66 ± 0.36^b^	1.02 ± 0.93			
LG2_VT/TI_ (a.u.)	1.00	0.08 ± 0.10^b^	0.07 ± 0.19^b^	0.16	**<0.0001**	0.19
	0.21	0.25 ± 0.20	0.33 ± 0.29			
	0.14	0.34 ± 0.31	0.56 ± 0.50			
LG3_VT/TI_ (a.u.)	1.00	0.06 ± 0.08	0.07 ± 0.18	0.3	**0.0349**	0.33
	0.21	0.16 ± 0.17	0.18 ± 0.28			
	0.14	0.10 ± 0.14	0.22 ± 0.29			
*T*_I_ 1 (s)	1.00	1.90 ± 0.55	2.20 ± 0.54	0.059	0.47	**0.011**
	0.21	1.98 ± 0.57	2.01 ± 0.40			
	0.14	1.84 ± 0.55	1.87 ± 0.56			
*T*_I_ 1+2 (s)	1.00	1.98 ± 0.63	2.10 ± 0.53	0.13	0.93	0.070
	0.21	2.06 ± 0.70	2.00 ± 0.49			
	0.14	1.97 ± 0.78	1.86 ± 0.55			
*T*_I_ 2 (s)	1.00	2.05 ± 0.70	2.00 ± 0.52	0.45	0.49	0.47
	0.21	2.14 ± 0.82	1.98 ± 0.58			
	0.14	2.09 ± 0.96	1.85 ± 0.57			
*T*_I_ 3 (s)	1.00	2.09 ± 0.74	1.92 ± 0.38	0.28	0.33	0.79
	0.21	2.03 ± 0.68	1.91 ± 0.50			
	0.14	2.18 ± 1.05	1.84 ± 0.53			
Breath hold duration (s)	1.00	9.9 ± 0.4^c^	10.2 ± 0.3	**0.005**	0.21	0.81
	0.21	9.9 ± 0.3^c^	10.2 ± 0.3			
	0.14	9.8 ± 0.4^c^	10.1 ± 0.3			
Recovery duration (s)	1.00	64.7 ± 3.7	63.9 ± 2.8	0.97	0.11	0.19
	0.21	64.8 ± 2.8	65.2 ± 3.6			
	0.14	64.9 ± 2.5	65.5 ± 3.6			
BL $S_{pO_{2}}$ (%)	1.00	99.8 ± 0.3^b^	99.7 ± 0.4^b^	0.28	**<0.0001**	0.71
	0.21	97.0 ± 2.1	95.9 ± 3.5			
	0.14	84.3 ± 4.9^b^	83.3 ± 4.5^b^			
Nadir $S_{pO_{2}}$ (%)	1.00	99.4 ± 0.6^b^	99.5 ± 0.6^b^	0.11	**<0.0001**	0.4
	0.21	95.5 ± 2.5	94.2 ± 3.7			
	0.14	80.6 ± 5.3^a^, ^b^	78.6 ± 3.8^b^			
Delta $S_{pO_{2}}$ (%)	1.00	0.35 ± 0.42^b^	0.25 ± 0.31^b^	0.88	**<0.0001**	0.86
	0.21	1.73 ± 0.82	1.71 ± 0.56			
	0.14	4.63 ± 2.97^a^, ^b^	4.89 ± 1.88^b^			

*Note*: *P*‐values are split between the sex effect, gas (oxygen) effect, and the interaction between sex and gas, from a two factor (repeated measures in gas; non‐repeated measures in sex) ANOVA across $F_{IO_{2}}$ (0.14, 0.21, 1.00) for each respective sex (males *n* = 18; females = 18). We bolded those hat were significant (*P* < 0.05)

^a^ Reduced sample size, *n* = 17.

^b^ Significantly different from $F_{IO_{2}}$ 0.21.

^c^ Significantly different from males. Breath holding variables: LG_VT_, loop gain using relative delta tidal volume of the 1st breath, average of the 1st and 2nd breath, 2nd breath, 3rd breath following breath hold (a.u.); LGVT/TI, loop gain using relative delta respiratory drive of the 1st breath, average of the 1st and 2nd breath, 2nd breath, 3rd breath following breath hold (a.u.); *T*_I_, time of inspiration (s) of the 1st breath; average of the 1st and 2nd breath, 2nd breath, 3rd breath following breath hold; BL $S_{pO_{2}}$, baseline $S_{pO_{2}}$ was measured prior to the start of each apnoea (%); Nadir $S_{pO_{2}}$, minimum $S_{pO_{2}}$ during recovery (%); Delta $S_{pO_{2}}$, the difference between nadir $S_{pO_{2}}$ and BL $S_{pO_{2}}$.

### 3.3 LG_VT_ and sex differences from breath holds

**FIGURE 2:**
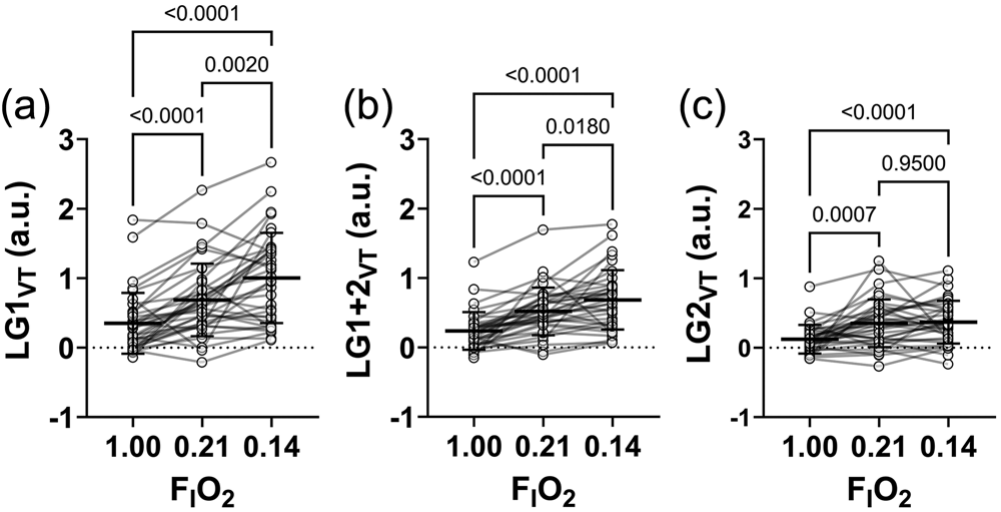
Comparison of different LG_VT_ ratios across three gas conditions ($F_{IO_{2}}$ 1.00, 0.21, 0.14). (a) LG1_VT_ across three gas conditions. (b) LG1+2_VT_ across three gas conditions. (c) LG2_VT_ across three gas conditions. Open circles denote individual participants with the connecting line to the same participant at different $F_{IO_{2}}$. The horizontal bar denotes the mean, and the error bars denote the SD. *n* = 36. a.u., arbitrary units; $F_{IO_{2}}$, fraction of inspired oxygen; LG_VT_, loop gain using relative delta tidal volume of the 1st breath, average of the 1st and 2nd breath, 2nd breath, 3rd breath following breath hold.

**FIGURE 3:**
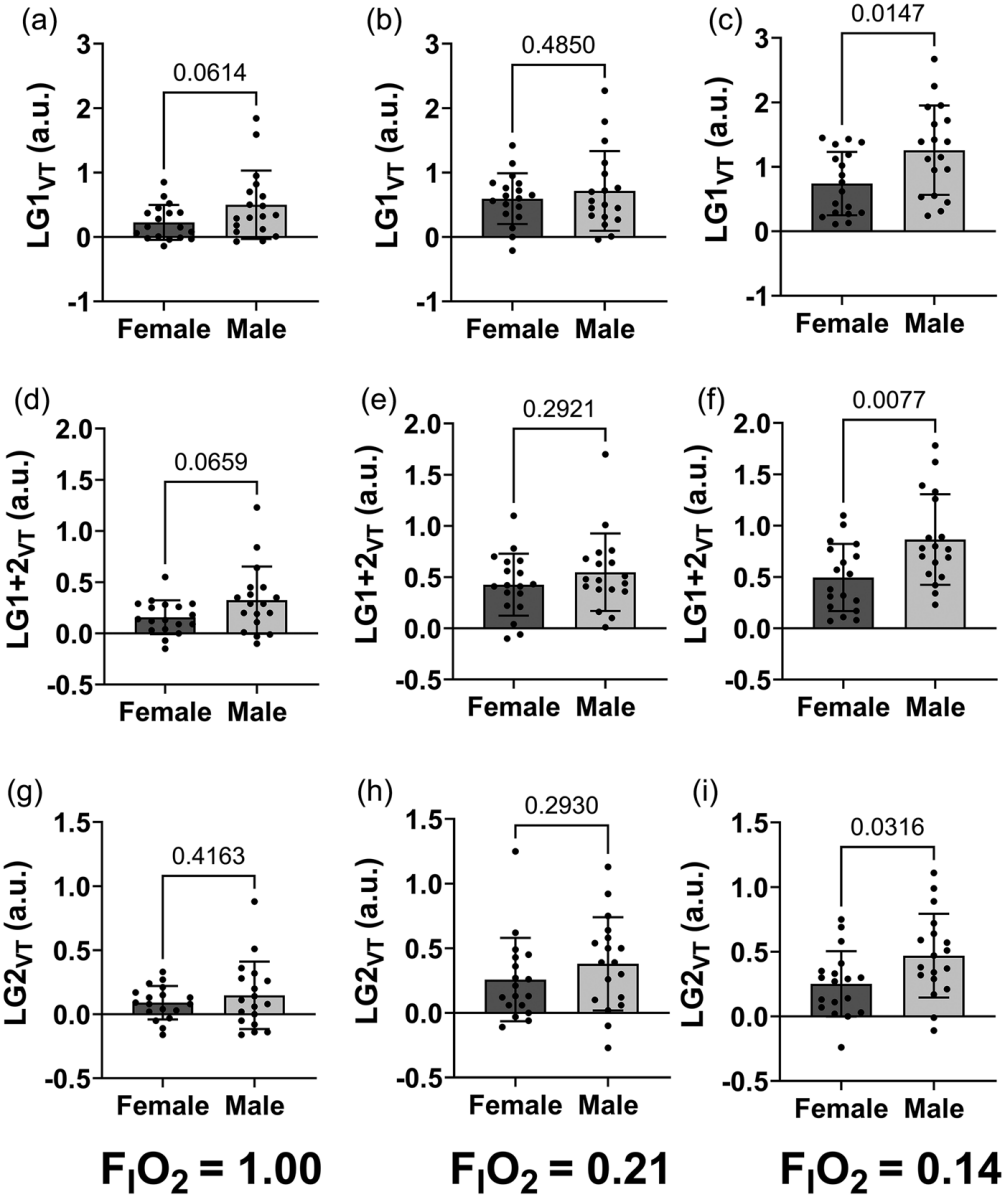
Sex difference comparison of different LG_VT_ ratios across different $F_{IO_{2}}$ (1.0, 0.21, 0.14). (a–c) Sex difference using LG1_VT_ at (a) $F_{IO_{2}}$ = 1.0, (b) $F_{IO_{2}}$ = 0.21, and (c) $F_{IO_{2}}$ = 0.14. (d–f) Sex difference using LG1+2_VT_ at (d) $F_{IO_{2}}$ = 1.0, (e) $F_{IO_{2}}$ = 0.21, and (f) $F_{IO_{2}}$ = 0.14. (g–i) sex difference using LG2_VT_ at (g) $F_{IO_{2}}$ = 1.0, (h) $F_{IO_{2}}$ = 0.21, and (i) $F_{IO_{2}}$ = 0.14. Colours denote different sexes; male = dark grey, female = light grey. Filled circles represent individual participants. The edges of boxes denote the mean, and the error bars denote the SD. Females *n* = 18; males, *n* = 18. a.u., arbitrary units; $F_{IO_{2}}$, fraction of inspired oxygen; LG_VT_, loop gain using relative delta tidal volume of the 1st breath, average of the 1st and 2nd breath, 2nd breath, 3rd breath following breath hold.

LG**_VT_** ratios (dependent on the breath following breakpoint used) were obtained for the group at all $F_{IO_{2}}$ levels (i.e., 1.0, 0.21 and 0.14) and are presented in Figure 2. There was an inverse oxygen‐dependent effect on LG**_VT_** magnitude for the three LG**_VT_** variables (LG1**_VT_**, LG1+2**_VT_** and LG2**_VT_**), with highest LG**_VT_** in hypoxia, moderate in normoxia and lowest in hyperoxia, as expected (*P *< 0.0281). However, there were no differences observed between normoxia and hypoxia for LG2**_VT_** (*P* = 0.96). LG**_VT_** ratios split by breath used, sex and gas condition across all $F_{IO_{2}}$ levels are presented in Figure 3. There were no differences observed between sexes with all LG**_VT_** ratios in both normoxia and hyperoxia (*P *> 0.075). Males had consistently higher LG**_VT_** than females in hypoxia with all three LG ratio calculations (Figure 3c,f,i; *P *< 0.025).

### 3.4 LG_VT/TI_ and sex differences from breath holds

**FIGURE 4:**
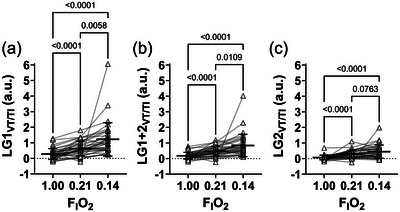
Comparison of different LG_VT/TI_ ratios across three gas conditions ($F_{IO_{2}}$ 1.00, 0.21, 0.14). (a) LG1_VT/TI_ across three gas conditions. (b) LG1+2_VT/TI_ across three gas conditions. (c) LG2_VT/TI_ across three gas conditions. Open triangles denote individual participants, with the connecting line to the same participant at different $F_{IO_{2}}$. The horizontal bar denotes the mean, and the error bars denote the SD. *n* = 36. a.u., arbitrary units; $F_{IO_{2}}$, fraction of inspired oxygen; LG_VT/TI_, loop gain using relative delta respiratory drive of the 1st breath, average of the 1st and 2nd breath, 2nd breath, 3rd breath following breath hold.

**FIGURE 5:**
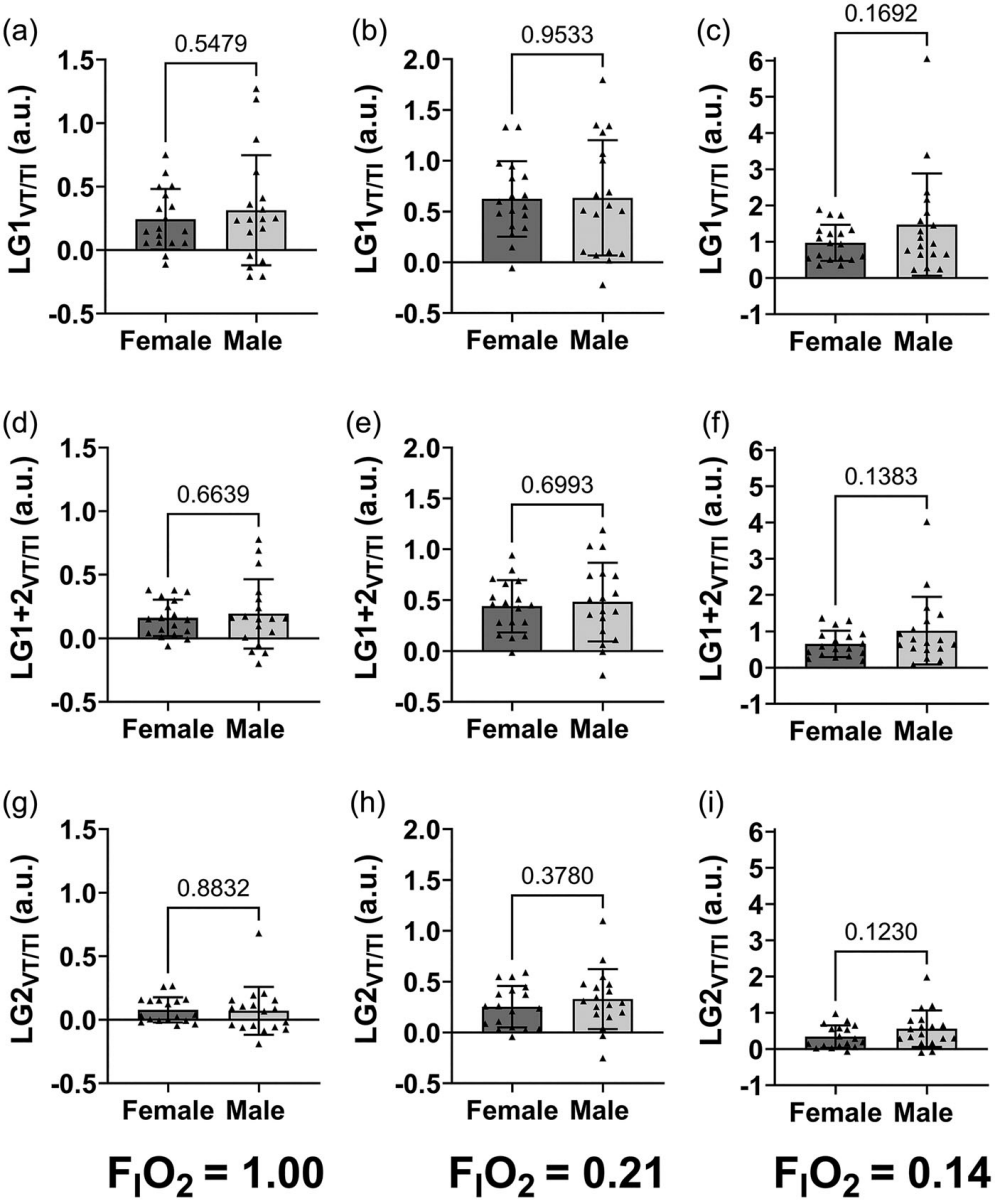
Sex difference comparison of different LG_VT/TI_ ratios across different $F_{IO_{2}}$ (1.0, 0.21, 0.14). (a–c) Sex difference using LG1VT/TI at (a) $F_{IO_{2}}$ = 1.0, (b) $F_{IO_{2}}$ = 0.21, and (c) $F_{IO_{2}}$ = 0.14. (d–f) Sex difference using LG1+2_VT/TI_ at (d) $F_{IO_{2}}$ = 1.0, (e) $F_{IO_{2}}$ = 0.21, and (f) $F_{IO_{2}}$ = 0.14. (g–i) Sex difference using LG1_VT/TI_ at (g) $F_{IO_{2}}$ = 1.0, (h) $F_{IO_{2}}$ = 0.21, and (i) $F_{IO_{2}}$ = 0.14. Colours denote different sexes; females = dark grey, males = light grey. Filled triangles represent individual participants. The edges of the boxes denote the mean, and the error bars denote the SD. Females *n* = 18; Males, *n* = 18. a.u., arbitrary units; $F_{IO_{2}}$, fraction of inspired oxygen; LG_VT/TI_, loop gain using relative delta respiratory drive of the 1st breath, average of the 1st and 2nd breath, 2nd breath, 3rd breath following breath hold.

LG_VT/TI_ ratios (dependent on the breath following the breakpoint used) were obtained for the group at all $F_{IO_{2}}$ levels (i.e., 1.00, 0.21 and 0.14) and are presented in Figure 4. There was a similar inverse oxygen‐dependent effect on LG_VT/TI_ magnitude for the three LG_VT/TI_ variables (LG1_VT/TI_, LG1+2_VT/TI_ and LG2_VT/TI_), with highest LG_VT/TI_ in hypoxia, moderate in normoxia and lowest in hyperoxia, as expected (*P *< 0.012). However, there were no differences observed between normoxia and hypoxia for LG2_VT/TI_ (*P* = 0.076). LG_VT/TI_ ratios split by breath used, sex and gas condition across all $F_{IO_{2}}$ levels are presented in Figure 5. There were no differences observed between males and females in all LG_VT/TI_ ratios across all gas conditions (*P *> 0.12).

## 4 DISCUSSION

We characterized a novel, voluntary end‐expiratory breath holds (EEBH) protocol to quantify LG during wakefulness, and the effects of three oxygen gas conditions: $F_{IO_{2}}$, 1.00 (hyperoxia; i.e., 100%), 0.21 (normoxia; i.e., 21%) and 0.14 (hypoxia; i.e., 14%), on 36 participants (18 males and 18 females). The principal findings were (a) an inverse oxygen‐dependent effect on LG_VT_ and LG_VT/TI_ magnitude across all three LG_VT_ ratio calculations; (b) a sex‐dependent effect only in hypoxia ($F_{IO_{2}}$ = 0.14) across all three LG_VT_ ratios (LG1_VT_, LG1+2_VT_ and LG2_VT_), where males had higher LG_VT_ than females; and (c) there were no sex differences seen in all LG_VT/TI_ ratios across all oxygen tensions. Our study (a) fully characterizes a novel EEBH protocol to assess LG during wakefulness and (b) highlights a distinct effect of sex on LG_VT_ in hypoxia. Together, these findings suggest that this EEBH protocol provides physiological insights which can be applied to further studies of CSA in the context of ascent and heart failure populations.

### 4.1 Effect of hypoxia and hyperoxia on LG

We found an inverse relationship between LG**_VT_** magnitude and oxygen availability across all LG**_VT_** ratios (Figure 3), which suggests that our novel EEBH protocol assesses differential chemosensitivity in the form of relative delta *V*_T_ (LG_VT_) with differential $F_{IO_{2}}$. Based on the short nature of the hypoxic stimulus (5 min) prior to the breath holds, Powell et al. (1998) indicates that the primary stimulus for the increased *V̇*_I_ is likely mediated by the decreased $P_{aO_{2}}$ and thus, the decrease in $P_{aO_{2}}$ stimulates and sensitizes the carotid body almost instantaneously for $P_{aO_{2}}$ and $P_{\mathrm{aC}O_{2}}$ (Dempsey et al., 2014; Lahiri et al., 2006). In addition, our end‐expiratory apnoeas were only ∼10 s; thus, the response to this stimulus was likely entirely peripherally mediated in response to the transient rise in $P_{\mathrm{aC}O_{2}}$ at the metabolic rate. Therefore, the increase in ventilation can be explained by the hyper‐additive effects of hypoxaemia plus the ventilatory response to an increase in $P_{\mathrm{aC}O_{2}}$ resulting from the breath holds. This cumulative effect that follows the breakpoint of each apnoea produces a powerful but transient respiratory response, from which we can use our LG calculations to quantify the overall $P_{\mathrm{aC}O_{2}}$/$P_{aO_{2}}$ sensitivity. Our findings are consistent with Messineo et al. (2018), as individuals with higher LG during wakefulness had a greater ventilatory chemoreflex response magnitude. The current study differs from Messineo et al. (2018), as we used a series of EEBHs in a background of different $F_{IO_{2}}$ (1.0, 0.21 and 0.14) compared to using transient inspiration of a 6% CO_2_–14% O_2_ gas mixture to simulate an apnoea. Our findings also suggest that this EEBH protocol can simulate a similar stimulus to CSA and can therefore provide an indication of how each individual will respond. For instance, in the case where a subset of our participants had a LG**_VT_** > 1.0 in hypoxia, this may cause high instability of the ventilatory control system, from which we hypothesize that they could develop sleep disturbances in the context of steady‐state acute or sustained hypoxia (CSA; Plataki et al., 2013). This application requires further experimental testing in the context of HA.

In hyperoxia compared to hypoxia, the opposite occurs, where the background of inspired hyperoxia blunts the carotid bodies’ sensitivity to changes in $P_{aO_{2}}$ and $P_{\mathrm{aC}O_{2}}$, such that the resulting rise in $P_{\mathrm{aC}O_{2}}$ during an EEBH produces a small increase in ventilation (i.e., low sensitivity), and thus low LG (Chowdhuri et al., 2010; Xie, 2012). Wellman et al. (2008) demonstrated that when obstructive sleep apnoea (OSA) patients were supplemented with hyperoxia during sleep, patients that had the greatest blunting effect also had high initial LG (>0.45), whereas it had negligible effects for patients with already low LG (< 0.3). In sleep‐focused studies (e.g., Bird et al., 2023; Wellman et al., 2008), LG is averaged based upon the entire night and therefore appears to be lower compared to our values obtained immediately following a series of voluntary breath holds. This difference is likely due to differences in calculations, as our method calculates LG using only the *V*_T_ component immediately following an apnoea, whereas during characteristic CSA, transient hyperventilation follows each apnoea as a response and therefore reduces LG with subsequent breaths as the individual recovers from the apnoea (i.e., stimulus). The current study confirms this finding, as with each LG**_VT_** ratio calculation based upon the breaths following the breakpoint, the ratios eventually reduce toward zero or below (i.e., not different from baseline prior to breath hold or smaller than baseline; Table 3).

We found that baseline *V̇*_I_ increased in both hypoxia and hyperoxia compared to normoxia (*P *< 0.0001), but these results are likely due to different underlying mechanisms (i.e., HVR vs. hyperoxic hyperventilation). There are two main hypotheses for the underlying mechanism for hyperoxic hyperventilation: (a) hyperventilation is mediated by an accumulation of reactive oxygen species (ROS) production acting on central respiratory neurons. Fernandes et al. (2021, 2020) indicated that the likely mechanism for hyperoxic hyperventilation is due to excessive production and accumulation of ROS in the brain, particularly in regions with high metabolic activity and oxygen consumption, which may either increase the sensitivity to CO_2_ or directly activate the central chemoreceptors, resulting in a relative hyperventilation state (Chen et al., 2018; Edwards et al., 2014). Therefore, this relative hyperventilation from hyperoxia appears to be a protective mechanism to decrease ROS in the brain (Dean et al., 2004; Mulkey et al., 2003). Or (b) that hyperventilation is the result of the Haldane effect, where CO_2_ accumulates in brainstem tissue that stimulates the central chemoreceptors as haemoglobin is fully saturated with O_2_ (Brugniaux et al., 2018). These two mechanisms are likly to occur simultaneously, but with differing time courses. As per the duration of the hyperoxic stimulus (5 min), it is unlikely to result in a rise in ROS great enough to be different from baseline and stimulate central chemoreceptors enough to increase *V̇*_I_. Leveque et al. (2022) demonstrated that while inspiring 100% O_2_, ROS increased by 8.6% at 30 min compared to baseline and peaks at 46.4% at 8 h compared to baseline. Aside from baseline ventilation from central factors, there remains high variability between individuals in peripherally mediated chemoreflex LG_VT_, and sex may play a role in affecting this variability.

### 4.2 Sex and chemoreflex sensitivity in hypoxia

During our EEBH protocol, females exhibited lower LG**_VT_** compared to males in a background of hypoxia (Figure 4c,f,i). Although sex differences in respiratory chemoreflex sensitivity have been reported previously (e.g., Caravita et al., 2015; Li et al., 2022; Lombardi et al., 2013), our novel protocol combines the calculations of LG**_VT_** during wakefulness that are influenced by hyperoxia, normoxia and hypoxia. Our findings are consistent with previous studies assessing sex differences in sustained hypoxia at HA, where Caravita et al. (2015) found higher resting *V̇*_I_ in males compared to females at 4559 m (10M/10F), which may indicate that males have a higher LG, although it was not calculated. Similarly, Bird et al. (2023) found that over a 10‐night acclimatization period to 3800 m, males experienced a progressive increase in LG during sleep, whereas females did not (12M/8F), which was related to more severe CSA in males compared to females. Clinical populations such as heart failure patients demonstrate similar findings to those of high‐altitude‐induced CSA. Gentile et al. (2022) found that females had decreased chemoreflex sensitivity to both hypercapnia and hypoxia alongside mild CSA, whereas Huang et al. (2024) confirmed this finding in females when compared against males with heart failure and CSA. Other studies have suggested that in acute hypoxia, there is greater chemoreceptor‐induced sympathetic activation in males compared to females, which could explain the larger ventilatory response observed in males (Bärtsch & Gibbs, 2007; Botek et al., 2018). It is also unknown if these sex differences persist following full acclimatization and prolonged stays at HA (Patrician et al., 2024). Longer duration studies in the context of HA ascent and residence are required to further assess sex differences and the time‐course of CSA development.

Despite our findings that males have higher LG**_VT_** than females when inspiring normobaric hypoxia, the physiological mechanisms that explain potential sex differences in breathing stability remain unclear. Throughout the literature, there remains a disparity regarding how sex hormones such as progesterone, oestrogen and testosterone influence ventilatory control. Some groups suggest they have negligible effects (Beidleman et al., 1999; Macnutt et al., 2012), but others that argue that they do in fact influence ventilatory control (Regensteiner et al., 1990). Receptors for circulating sex hormones are also known to be in both the carotid body and brainstem chemoreceptors, and therefore it is difficult to assess the specific influence of sex hormones on ventilation and/or respiratory sensitivity to blood gas challenges in different conditions (Behan & Wenninger, 2008). Progesterone is known to have a stimulatory effect on resting ventilation, by increasing the chemosensitivity to prevailing CO_2_, with the effect exacerbated during pregnancy (LoMauro & Aliverti, 2015). Additionally, during hypoxic resting conditions, progesterone and oestrogen mediate higher peripheral chemosensitivity (Tatsumi et al., 1997). Richalet et al. (2020) demonstrated that in hypobaric hypoxia, the HVR during exercise in premenopausal females was higher in the early to mid‐luteal phase compared to early follicular phase; however, they found no difference compared to males. While this study benefited from a very large sample size, unfortunately they did not confirm the ovarian phase via hormonal analysis. These findings from Richalet et al. (2020) confirm that circulating sex hormones in females have no effect on the HVR compared to males. In the present study, we tested females across their self‐reported ovarian cycle, with six females being in their follicular stage, seven during the luteal phase, one during ovulation and four were unknown. In addition, five were using hormonal contraceptives and 13 were not. In addition, Richalet et al. (2020) found no effect of hormonal or oral contraceptive on ventilatory responses to hypobaric hypoxia in both pre‐ and post‐menopausal females. Circulating hormones like progesterone and oestrogen tend to receive more attention compared to testosterone because they are characteristic of ovarian cycling and have negligible effects in males. Ahuja et al. (2007) demonstrated that when premenopausal women without contraception (11F) were administered testosterone, their chemoreflex sensitivity to $P_{\mathrm{aC}O_{2}}$ increased, but only in hyperoxia (100% O_2_), having no effect in hypoxia (8% O_2_, balance N_2_). This finding confirms that sex hormones like testosterone do increase chemoreflex sensitivity, but does not explain the sex‐difference in hypoxia like the present study and those that demonstrate a sex difference at HA (e.g., Bird et al., 2023). Based on these considerations, the sex differences we demonstrate here only in hypoxia across the three LG_VT_ ratio calculations are unlikely due to variation in cycling sex hormones. This is because we had a relatively large sample size (18M/18F), so individual differences in the concentration of sex hormones are expected to average out across the sample size, and thus this finding likely represents an intrinsic sex difference in LG_VT_ between young adult males and females.

### 4.3 Sex differences in respiratory drive and inspiratory time in hypoxia

There were no sex differences found in any of the LG_VT/TI_ variables across all oxygen tensions. LG_VT/TI_ ratios involve the standardization of mean inspiratory flow rate (*V*_T_/*T*_I_) as an index of central respiratory drive (Figure 5; *P *> 0.5; Georgopoulos et al., 2024; Morgan et al., 2014). These findings differ from LG_VT_ as females had a blunted *V*_T_ response to BH in hypoxia (Figure 3c,f,i) as *T*_I_ is modulated differently between females and males (Table 3). Our findings align with Neder et al. (2003), where females had a shorter *T*_I_ compared to males (although not significant), but there were no sex differences observed in *V*_T_/*T*_I_ for any given breath following the BH.

The lack of sex differences for LG_VT/TI_ likely stems from differences in central respiratory centres, particularly regions that regulate the rate and depth of the breath that augment the structural dimensions and respiratory mechanics of females and males. Central respiratory centres work together to regulate involuntary and voluntary/behavioural control of breathing (Del Negro et al., 2018; Khalilpour et al., 2024; Richter & Smith, 2014), whereas central respiratory drive is dependent on the intensity of the output signals to the respiratory muscles and from regions that regulate *T*_I_ (Costa et al., 2017; Kam et al., 2013; Mador & Tobin, 1991). Hudson et al. (2024) found that females distribute central inspiratory drive signals more evenly across the first five parasternal intercostal muscles and had a shorter latency as compared to males, who had a longer latency of the neural drive signal to the fourth and fifth parasternal intercostal muscles (De Troyer et al., 2003). Since females distribute the central respiratory drive signal more evenly across the rib cage, this is in line with Mitchell et al. (2018), as they found that females had greater activation of extra‐diaphragmatic muscles, particularly the scalene and sternocleidomastoid muscles, for the same relative ventilation compared to males. These findings suggest that females modulate central respiratory drive differently from males to the respiratory muscles, and the structural dimensions and mechanics of breathing may further augment these differences.

From a structural and respiratory mechanics perspective, females have smaller pharyngeal cross‐sectional areas, smaller airways and higher airway resistance, which leads to an increased work of breathing, even when standardized to height (Brooks & Strohl, 1992; LoMauro & Aliverti, 2018; Peters et al., 2021; Sheel et al., 2016). Findings from Bellemare et al. (2006, 2003) demonstrate that females have smaller ribcages after standardizing for height and having a shorter diaphragm length. Due to the altered structural difference in the ribcage and the diaphragm, females need to maximize their mechanical advantage of the central inspiratory drive signal given the altered differences compared to males (Butler, 2007; Hudson et al., 2011; LoMauro & Aliverti, 2018). However, since females are already maximizing their mechanical advantage, any further augmentation would be ineffective. This may be seen during hyperinflation, where females’ capacity to further stretch their lungs is reduced as lung compliance decreases exponentially nearing total lung capacity, which may parallel the limited *V*_T_ responses to EEBH in hypoxia (McClaran et al., 1998; Sheel & Guenette, 2008). Although diaphragm lengths were not measured in the present study, we can infer that a shorter diaphragm length in females could be observed at all ranges of lung inflation, favouring a shorter *T*_I_ (Bellemare et al., 2003; Neder et al., 2003). All of these findings explain why the sex difference noted in LG_VT_ ratios were absent in LG_VT/TI_ ratios, because males had greater *V*_T_ responses in hypoxia, but did not change *T*_I_, whereas females had minimal changes in *V*_T_ and a shorter *T*_I_ in hypoxia but were not significantly different compared to males (Table 3).

### 4.4 Limitations

A number of limitations are important to consider in interpreting our data. It is important to note that we did not quantify $P_{aO_{2}}$ or $P_{\mathrm{aC}O_{2}}$ before and after EEBHs directly, as we wanted to maximize our sample size and reduce participant burden with a short protocol, without adding the invasiveness of arterial blood draws. Measuring end‐tidal blood gases is a reliable surrogate to arterial blood gases, but with mild effects of tracheal and alveolar dead space (Manferdelli et al., 2023; Satoh et al., 2015; West et al., 2019). There can be high variability in the magnitude of $P_{\mathrm{aC}O_{2}}$ increases from a breath hold between participants, as the amount of $P_{\mathrm{aC}O_{2}}$ produced is influenced by individual baseline blood gas levels and metabolic rate (Trembach & Zabolotskikh, 2017). We did not clamp $P_{\mathrm{ETC}O_{2}}$ during baseline in each steady‐state oxygen trial, and given the short duration of breath holds in the present study (10 s), nor did we measure expired gases prior to each breath hold or following breakpoint (e.g., Bruce et al., 2016; Marullo et al., 2022). The relative hypocapnia in the background of hypoxia may affect the background steady‐state chemoreflex activation compared to isocapnic (i.e., normocapnic) conditions (Becker et al., 1996). However, since this study has potential application to HA and heart failure induced alterations in LG and resulting CSA, isocapnic conditions would not be representative, as progressive hyperventilation occurs, thus creating steady‐state hypocapnic conditions (Hernandez & Patil, 2016; Khoo, 2000; see Table 1).

### 4.5 Applications and future directions

Historically, there have been many ways that respiratory chemoreflexes are quantified. For instance, hypercapnic ventilatoty respose (HVR/HCVR) magnitude can be assessed in a laboratory context (Teppema & Dahan, 2010), which may include methods such as dynamic end‐tidal forcing (e.g., Steinback & Poulin, 2007), rebreathing (e.g., Duffin, 2011) and transient gas‐perturbation tests (e.g., Pfoh et al., 2016). However, there remain many caveats associated with each of these methods, particularly (a) feasibility in fieldwork or laboratory contexts, (b) high within‐participant variability (e.g., Borle et al., 2017; Pfoh et al., 2016), (c) cofounding cardiovascular and cerebrovascular variables in response to blood gas challenges (e.g., Hoiland et al., 2015; Pfoh et al., 2016), and (d) differential responses when compared between tests (e.g., Pfoh et al., 2016; Steinback & Poulin, 2007). In terms of clinical contexts, administering peripheral chemoreceptor sensitivity tests using hypoxic gas challenges may be potentially dangerous or uncomfortable for patients experiencing sleep apnoea, heart failure and chronic obstructive pulmonary disease, with the risk of significant oxygen desaturation during the tests (e.g., Jura et al., 2022; Narkiewicz, Pesek et al., 1999; Narkiewicz, van de Borne et al., 1999). However, the short voluntary breath hold protocol established here to assess LG may have utility in clinical contexts, because chemoreflex hypersensitivity has been identified as one of the main mechanisms behind many cardiorespiratory disorders such as heart failure (Marcus et al., 2014; Stickland et al., 2007), CSA (Mansukhani et al., 2015) and OSA (Trombetta et al., 2013). This breath‐hold protocol may also predict the severity of CSA in heart failure patients (e.g., Solin et al., 2000). By assessing chemoreflex sensitivity in the form of LG using a simple voluntary EEBH protocol, there may be utility in applying this LG metric to assessing or predicting CSA in the context of HA acclimatization and/or heart failure patients.

### 4.6 Conclusion

We characterized the utility of a series of voluntary EEBHs during wakefulness to quantify LG_VT_ and determine the potential gas‐dependent and sex‐specific differences in LG_VT_ across three types of inspired oxygen levels (100%, 21%, and 14%). We found that (a) there was an inverse oxygen‐dependent effect on all three LG_VT_ ratio calculations (LG1_VT_, LG1+2_VT_, and LG2_VT_) following break point and (b) for all three LG_VT_ ratio calculations, males had a larger magnitude LG_VT_ response compared to females, but only in a background of hypoxia. We also found that (c) there was an oxygen‐dependent effect on three LG_VT/TI_ ratio calculations (LG1_VT/TI_, LG1+2_VT/TI_ and LG2_VT/TI_), but there were no differences between males and females in all of the LG_VT/TI_ ratios in all oxygen tensions. These findings highlight the sex‐specific differences in chemoreflex LG_VT_ in hypoxia using a series of EEBH as a safe and feasible surrogate for the HVR/HCVR. These data may have utility in understanding high variability of chemoreflex LG, and its effect on CSA with ascent to high altitude and in heart failure populations.

## AUTHOR CONTRIBUTIONS

Conception and design of the work: Trevor A. Day. Acquisition, analysis, or interpretation of data for the work: all authors. Drafting the work or revising it critically for important intellectual content: all authors. In addition, all authors approved the final version of the manuscript and agree to be accountable for all aspects of the work in ensuring that questions related to the accuracy or integrity of any part of the work are appropriately investigated and resolved. All persons designated as authors qualify for authorship, and all those who qualify for authorship are listed.

## CONFLICT OF INTEREST

None declared.

## Data Availability

The de‐identified, numerical data that support the findings of this study are available from the corresponding author upon reasonable request from a qualified researcher.
